# Training in women soccer players: A systematic review on training load monitoring

**DOI:** 10.3389/fpsyg.2022.943857

**Published:** 2022-07-29

**Authors:** Júlio A. Costa, Vincenzo Rago, Pedro Brito, Pedro Figueiredo, Ana Sousa, Eduardo Abade, João Brito

**Affiliations:** ^1^Portugal Football School, Portuguese Football Federation, Oeiras, Portugal; ^2^Faculty of Health Sciences and Sports, Universidade Europeia, Lisbon, Portugal; ^3^Research Center in Sports Sciences, Health Sciences and Human Development, University of Maia, Maia, Portugal; ^4^Research Center in Sports Sciences, Health Sciences and Human Development, Vila Real, Portugal; ^5^Centro de Investigação em Desporto, Educação Física, Exercício e Saúde, Universidade Lusófona, Lisbon, Portugal

**Keywords:** workload, global positioning systems, heart rate, rating of perceived exertion, female athletes

## Abstract

**Objective:**

The present systematic review aimed to provide an overview of training load (TL), along with their responses, monitoring during training sessions in highly trained and elite adult women soccer players.

**Data source:**

Electronic databases searches (PubMed, Scopus, Web of Science and Ebsco) for relevant studies published in peer-reviewed journals were conducted, and eligibility criteria were based on the PICOS model in accordance with PRISMA guidelines.

**Study selection:**

Studies were considered as follows: (a) highly trained and elite adult (>18 years) women’s soccer players; (b) continuous (minimum 1-week duration) TL monitoring in the context of the team routine; (c) TL collected from entire training session. Methodological qualitative assessments and risk of bias criteria were used for judging the studies.

**Data extraction:**

A total of 1,163 studies were identified, and 16 were included. The selected studies were fully screened to extract the population characteristics; the number of players; a type of study design; region where the study was performed; the main findings.

**Data synthesis:**

Accumulated external TL (ETL) during the pre-season was positively correlated to enhanced adaptations in intermittent exercise capacity. Daily ETL was negatively correlated to next-day self-reported fatigue and muscle soreness. Daily internal TL (ITL) was negatively correlated to post-session sleep duration and sleep efficiency. One study showed that higher accumulated player load and total distance were associated with injury.

**Conclusion:**

Information about TL during training sessions in women soccer players is very sparse, and it is currently very difficult to consider evidence-based practices for training sessions in highly trained and elite adult women soccer players. Moreover, the dose–response relationships between TL and training outcome (e.g., fatigue, training adaptations and injuries) need to be further explored to understand the optimal training stimulus to enhance performance outcomes while preserving player health.

## Introduction

The popularity of women’s soccer has markedly increased over the last 10 years (Randell et al., 2021). Alongside, the professionalism has also increased, and current elite players might be exposed to higher training and competitive demands than before, possibly having implications for both performance and health (Datson et al., 2014). However, a recent bibliometric analysis noted that studies investigating elite women soccer players account for just around 15% of all soccer research published (Kirkendall and Krustrup, 2021), while several match- and training-related topics specifically dedicated to women’s soccer are still in need of greater attention.

In women’s soccer, as well as the male equivalent, it is incumbent that coaches and support staff optimize the health, well-being, and performance of the players. But in contrast to men’s soccer, and largely due to the increased female participation, science has struggled to keep pace with the demand for evidence-based studies to inform practice (Okholm Kryger et al., 2021). In a recent narrative review (Randell et al., 2021), it has been reported that the most popular publication topics related to women’s soccer are sports medicine, physiological, health and performance outcomes.

Within this context, a better understanding of the training process in elite women soccer players is vital to define appropriate strategies that may contribute to enhance performance, accelerate recovery, and reduce injury risk. Collectively, training responses, fatigue and injury risk can be described as training outcomes. However, the interplay between training load (TL), fatigue and injury risk is still unclear (Jaspers et al., 2017). Moreover, to the best of our knowledge, this information is yet to be reviewed in women soccer players.

Recent systematic reviews conducted in men’s and women’s soccer describing published TL practices (including data collection and interpretation) revealed that information about women’s soccer is very sparse (Rago et al., 2019a,b; Torres-Ronda et al., 2022). These reviews considered methods to collect and interpret TL, such as wearable technology incorporating global positioning systems (GPS) to quantify the external TL (ETL; Rago et al., 2019a; Torres-Ronda et al., 2022), the rating of perceived exertion (RPE) and the session-RPE (s-RPE: perceived intensity multiplied by the exposure time) to subjectively quantify internal TL (ITL; Rago et al., 2019b; Torres-Ronda et al., 2022). Quantified ITL methods (such as heart rate, HR) have also been included.

Therefore, considering the scarce literature and the aforementioned potential advantages associated with a better understanding of training, the present systematic review aimed to provide an overview of ETL and ITL monitoring during training sessions in highly trained and elite adult women soccer players, with a special focus on fatigue, training adaptions and injuries.

## Methods

This systematic review was conducted according to the Preferred Reporting Items for Systematic Reviews and Meta-Analyses (PRISMA) 2020 guidelines (Page et al., 2021). The protocol was registered at the International Platform of Registered Systematic Review and Meta-analysis Protocols (INPLASY 2021120038).

### Eligibility criteria

For the current systematic review, eligibility criteria were based on the PICOS model in accordance to the PRISMA statement (Shamseer et al., 2015) and other systematic reviews published regarding team routines in soccer (Rago et al., 2019a,b; Torres-Ronda et al., 2022); *Study design:* observational; *Participants and setting*: highly trained and elite adult (>18 years) women’s soccer players (Mckay et al., 2022) (i.e., players competing at the international leagues/tournaments; players competing in national and/or state leagues/tournaments; individuals on a national team); *Interventions*: continuous TL monitoring during training sessions in the context of the team routine; *Outcomes*: TL collected from entire training session; *Timing*: minimum 1-week duration of training.

### Literature search strategy

A systematic search was conducted in PubMed, Scopus, Web of Science and EBSCO combining the following groups of key words in the title, abstract or key words: (women OR female) AND (football OR soccer) AND (elite OR professional OR top-level OR highly trained) AND (load OR intens* OR volume OR training OR monitor* OR quantif* OR speed OR acceleration OR heart rate OR subjective OR rat* OR perce* effort OR exertion) AND (GPS OR “global positioning system” OR LPS OR “local positioning system” OR “time motion” OR physiolog*) AND (fatigue OR adaptations OR performance OR testing OR injury) AND NOT (“American Football” OR “Australian Football” OR AFL). The search was restricted to English peer-reviewed journals from 2000 to April 2022. Then, we further searched the relevant literature using the ‘related citations’ function of PubMed and by scanning reference lists of each article.

### Study selection

All records were exported to EndNote (Clarivate Analytics, Philadelphia, PA, United States) and duplicates were removed by using an automated tool and checked manually. Two authors (JC and PB), independently performed the searches and reviewed the studies. In case of disagreement, inclusion was discussed, and unresolved discrepancies were settled by a third reviewer (JB).

The articles were considered if published on-line regardless of the publication status. To investigate continuous TL monitoring during training sessions, we included articles with a minimum of 1-week duration, respective of sex and study focus (e.g., studies reporting descriptive data of TL without studying its effects were included). Articles were excluded if: the participants (a) were not all highly trained and elite adult women soccer players (e.g., mixed samples including highly trained/elite adult elite and non-highly trained and non-elite players); (b) were aged under 18; (c) were not monitored longitudinally over a minimum of a 1-week duration, or five sessions if the duration was not stated (friendly matches were considered training sessions), to consider continuous monitoring practices (Rago et al., 2019a,b); (d) the articles did not report any TL indicators as described by Halson (2014); single drills were monitored rather than the entire training session, or the article focused on the comparison between a specific drill and match demands; (e) data from training sessions were not reported; and (f) the articles were editorials or reviews. In the event of ambiguity in the title or abstract, the full-text article was checked for verification by two independently authors (JC and PB). The full-text articles of the remaining studies were then downloaded and archived. The references of the selected articles were then screened to identify any potentially relevant articles not identified by the original search. Afterward, the corresponding authors of the selected articles were contacted (via e-mail or social media) requesting missing information. When contacted, the authors were informed about the purpose of the study and no conflict of interest was declared. Information provided by the authors was labeled within the tables.

### Data extraction and management

All data on study characteristics and outcomes were extracted from all included studies by one author (PB) and subsequently reviewed by other author (JC).

The selected studies were fully screened to extract the population characteristics (i.e., age and competitive level); number of player’s and training sessions; type of study design; region where the study was performed; training period and duration; the monitoring TL method used; and the synthesis of main findings. If reported, TL data and the correlations between TL and training outcomes (fatigue, training adaptations, and injury risk) were also extracted. Only data exclusively related to training sessions have been extracted (i.e., match data have been excluded).

### Quality assessment of included studies

Two independent authors (JC and EA) assessed the quality of the included studies. The quality score of each study was based on a 16-item checklist adapted from a previous systematic review in soccer (Sarmento et al., 2018). Publications were evaluated based on: (1) clarity of purpose; (2) relevance of background literature; (3) appropriateness of the study design; (4) study sample; (5) sample size justification; (6) informed consent (if any); (7) outcome measures – reliability; (8) outcome measures – validity; (9) detailed method description; (10) significance of results reporting; (11) analysis methods; (12) practical importance; (13) description of drop-outs (if any); (14) appropriately conclusions; (15) practical implications; (16) study limitations. A binary scale was used to score these items (1 = yes; 0 = no), except for items (6) and (13), which could also be classified as not applicable (n/a). After that, a percentage score was calculated for each study by summing the scores of all items and dividing that by the maximum score the study could achieve. The publications’ quality score was classified as: (1) low methodological quality for scores ≤ 50%; (2) good methodological quality for scores between 51% and 75%; and (3) excellent methodological quality for scores >75% (Supplementary Table 1).

### Risk of bias

Two independent authors (VR and PB) underwent a calibration exercise, and then assessed the risk of bias of TL monitoring studies in women’s soccer (observational designs) using the Risk of Bias Assessment tool for Non-randomized Studies (RoBANS) tool (Kim et al., 2013; Supplementary Table 2). Conflicts were resolved through discussion among the pair of reviewers or through consultation with a third reviewer (JB).

## Results

### Study selection and study characteristics

**FIGURE 1:**
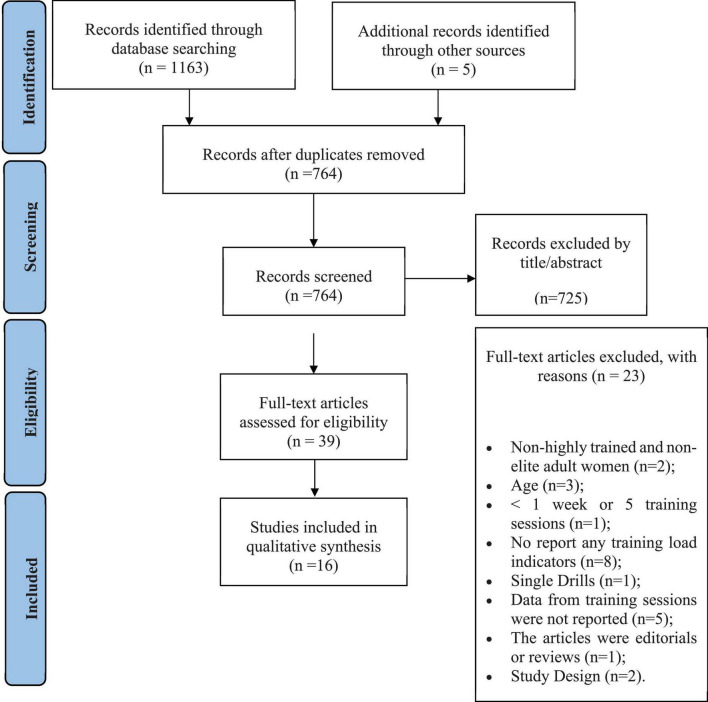
Preferred reporting items for systematic reviews and meta-analyses (PRISMA) diagram of the literature search results.

Initially, *1163* records were identified. After removing duplicates, screening the titles and full texts, 16 original articles met the inclusion criteria (Figure 1).

The selected articles were published from January 2000 to April 2022. Thirteen studies were conducted *across* various national leagues at the club level (Mara et al., 2015a,b; (Costa et al., 2018a,b, 2019b,2019c, 2021a,b; Clemente et al., 2019; Douchet et al., 2021; Fernandes et al., 2021; Romero-Moraleda et al., 2021; Xiao et al., 2021), while three studies were conducted in a National team setting (Scott and Lovell, 2018; Costa et al., 2019a; Doyle et al., 2021). The selected studies were predominantly conducted during periods lasting 1 to 25 weeks (Mara et al., 2015a,b; Costa et al., 2018a,2019b,2019c,2021a,2021b; Clemente et al., 2019; Douchet et al., 2021; Fernandes et al., 2021; Romero-Moraleda et al., 2021; Xiao et al., 2021) or during international tournaments lasting 10 to 21 days (Scott and Lovell, 2018; Costa et al., 2019a; Doyle et al., 2021). Only one study considered more than one entire season (i.e., three seasons), lasting 15 months (i.e., 5 months each season) (Xiao et al., 2021). A detailed description of the selected studies is reported in Table 1.

**TABLE 1:** Studies quantifying training load in highly trained and elite adult women’s soccer players over a minimum of 1 week (*n* = 16), and respective quality score.

Reference	Population characteristics (age and level)	Number of players/Sessions /Region	Type of study design	Period/Duration	Monitoring training load method	Synthesis of main findings	Quality score (%)
Clemente et al., 2019	23.6 ± 4.8/National league	89/6–7 training sessions + non-official match/Germany and Portugal	Prospective cohort study	Pre-season/5 weeks	GPS	Small-to-moderate intra-week load variance and no significant changes in weekly load variances based on total distance and sprinting distance. Significant differences were found between training days considering the duration (*p* = 0.011), walking distance (*p* = 0.017), running distance (*p* = 0.004), player’s load (*p* = 0.040) and number of sprints (*p* = 0.006).	93.3%
Costa et al., 2018a	21.4 ± 2.1/National league	18/8/Portugal	Single-group longitudinal	Competitive/3 weeks	HR, s-RPE	TRIMP, HR_*mean*_ and s-RPE varied throughout the week.	93.3%
Costa et al., 2018b	21.5 ± 0.9/National league	11/3/Portugal	Single-group observational	Competitive/1 week	HR, s-RPE	Descriptive values only.	86.7%
Costa et al., 2019a	25.2 ± 3.1/National Team	20/6/Portugal	Single-group observational	Competitive/9 days	GPS, s-RPE	Despite the significant day-to-day variations in TD, HSR and s-RPE, these variables were not correlated to post-session total sleep time, sleep efficiency and lnRMSSD.	93.3%
Costa et al., 2019b	21.6 ± 2.3/National league	17/18/Portugal	Single-group longitudinal	Competitive/6 weeks	HR, s-RPE	TRIMP, HR_*mean*_ and s-RPE were lower on night training days, and higher during away matches.	93.3%
Costa et al., 2019c	21.4 ± 2.1/National league	17/18/Portugal	Single-group observational	Competitive/6 weeks	HR, s-RPE	s-RPE was largely correlated with TRIMP (*r* = 0.74–0.82).	93.3%
Costa et al., 2021a	21.8 ± 2.6/National league	16/Nr/Portugal	Single-group longitudinal	Pre-season/4 weeks	s-RPE	Players improved aerobic fitness, along with increased 24-h cardiac vagal activity. The relative changes in HF24h and HF index were largely correlated with improvements in the distance covered during the Yo-Yo IR1 (*r* = 0.68 and *r* = 0.56; respectively).	100%
Costa et al., 2021b	20.6 ± 2.3/National league	34/8/Portugal	Single-group longitudinal	Competitive/14 days	HR, s-RPE	s-RPE and TRIMP were slightly to moderately correlated with sleep duration and sleep efficiency (*r* = −0.43 to −0.17) but not with HR variability parameters (lnRMSSD, lnLF, lnHF).	93.3%
Douchet et al., 2021	24.2 ± 2.3/National league	12/6/France	Single-group observational	Competitive/2 weeks	GPS, HR, s-RPE	Total number of accelerations and decelerations were greater during the heavy week than during the low week (*p* < 0.001). The mean HR%, total distance, m⋅min^–1^, RPE, sRPE and the Hooper Index were significantly greater during the heavy week. There were significant differences (*p* < 0.001) between the start and the end of the heavy week for Sleep, Fatigue, and DOMS.	93.3%
Doyle et al., 2021	24.2 ± 4.4/National Team	18/6/Ireland	Single-group observational	Competitive/1 week	GPS, s-RPE	Training load peaked on MD-5 as all variables significantly increased in comparison to MD-6 and MD-7. A significant decrease in volume and intensity was evident on MD-3 due to reductions on TL (*P* = 0.001, *r* = 0.60), TD (*P* = 0.001, *r* = 0.60), VHSR (*P* = 0.001, *r* = 0.61) and SPD (*P* = 0.00, *r* = 0.62). Significant difference in VHSD, SPD and SP between position on MD-2.	93.3%
Mara et al., 2015b	Nr/National league	17/90/Australia	Single-group longitudinal	Pre-season and competitive/18 weeks	GPS	Players covered greater TD and HSD during pre-season compared to early season, and then decreased in late season. TL was not correlated with fatigue, muscle soreness, sleep time, and changes in sprint performance. TD, HSR and accelerations were correlated to changes in Yo-Yo IR2 performance from pre-season to early season (*r* = 0.55–0.70).	86.7%
Romero-Moraleda et al., 2021	26.5 ± 5.7/National league	18/20/Spain	Single-group longitudinal	Competitive/5 months	GPS, s-RPE	The EL and the IL from official matches were higher compared to training sessions (*p* < 0.05; effect size [ES]:0.6–5.4). The training sessions MD + 1 and MD-2 showed the lowest EL and IL values. During MD, significant differences in EL and IL were noted between playing positions, although not during training sessions.	92.9%
Scott and Lovell, 2018	21.9–39.5/National Team	22/16/Australia	Single-group longitudinal	Competitive/21 days	GPS, HR, RPE	Irrespective of the quantification method adopted for HSD and very HSD (fixed or individualized speed zones), negative small correlations were observed with fatigue and soreness (*r* = −0.25 to −0.14).	92.9%
Xiao et al., 2021	Nr/National league	65/Nr/United States	Prospective cohort study	Competitive/3 seasons	GPS	There were no significant differences in player load, total distance, or high-speed distance ACWR between injured and non-injured players, regardless of the type of ACWR calculation (EWMA and Simple moving average). The prior 2-week, 3-week, and 4-week accumulated player loads were significantly higher for injured players. Similarly, the prior 2-week, 3-week, and 4-week accumulated total distances were significantly higher for injured players.	100%
Fernandes et al., 2021	24.1 ± 2.7/National league	19/30/Portugal	Single-group longitudinal	Competitive/10 weeks	RPE	Associations were found between Hooper Index categories and s-RPE like stress or fatigue (0.693, *p* < 0.01), stress or DOMS (0.593, *p* < 0.01), stress or s-RPE (0.516, *p* < 0.05) and fatigue or DOMS (0.688, *p* < 0.01). No differences were found in playing position or status when considering in-season IL and perceived well-being variation.	93.3%
Mara et al., 2015a	23–30/National league	8/5/Australia	Single-group observational	Pre-season/1 week	GPS	No differences between match and training days (*p* = 1.00) in mean total energy expenditure, however, significant differences were found between individual training sessions (*p* = 0.001–0.035). Significant differences with large effect sizes between friendly match and training sessions were found for total distance and HSD, but not sprinting distance, acceleration count or deceleration count.	93.3%

HR, heart rate; HSD, high-speed distance; lnRMSSD, natural logarithm of square root of the mean of the sum of the squares of differences between adjacent NN intervals; lnLF, natural logarithm of low-frequency; lnHF, natural logarithm of high-frequency; s-RPE, session-rating of perceived exertion; Yo-Yo IR1, Yo-Yo Intermittent Recovery – Level 1; Yo-Yo IR2, Yo-Yo Intermittent Recovery – Level 2; TD, total distance; TL, training load; TRIMP, training impulse; DOMS, delayed onset muscle soreness; SWA, SenseWear Mini Armbands; EL, external load; IL, internal load; MD, match-day; ACWR, acute-to-chronic workload rations; EWMA, exponentially weighted moving averages; Nr, not reported.

### Quality assessment of the studies

The mean methodological quality score for the 16 selected articles was 93.3%, with two articles achieving the maximum score of 100% (Table 1). Among the nine selected studies, the quality score ranged between 86.7 and 100%. All articles achieved an overall rating score of >75% (excellent methodological quality). Potential limitations found were mainly related to the lack of explicit justification for the sample size (criterion 5) and the absence of clear acknowledgment of study limitations (criterion 16).

### Risk of bias

**FIGURE 2:**
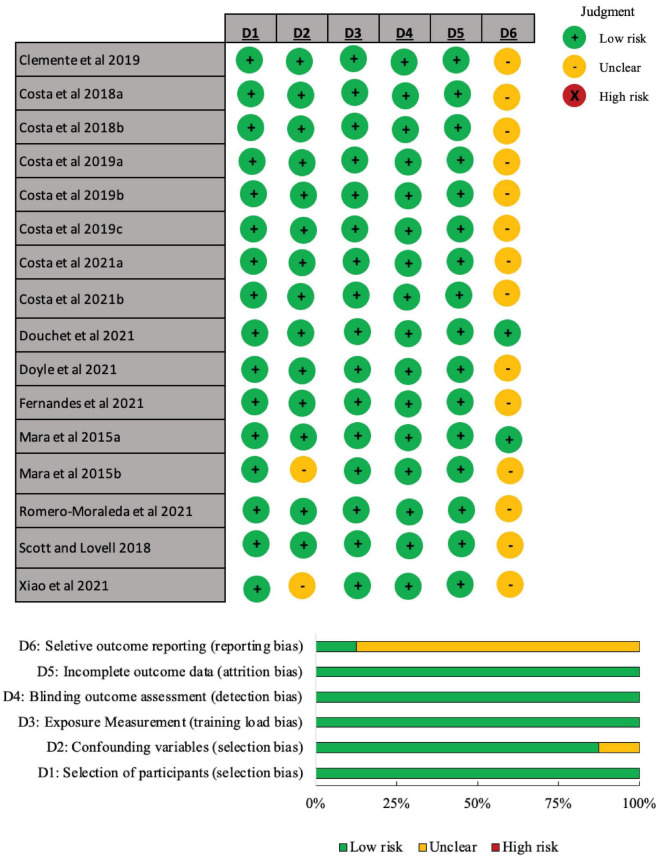
Risk of bias judgments for training load monitoring in highly trained and elite adult women’s soccer studies through RoBANS.

The “selection of the participant,” “exposure measurement,” “blinding outcome assessment” and “incomplete outcome data” were judged as low risk of selection of bias in 100% of the studies (Figure 2). For most of the studies (*n* = 14), the “confounding variables” domain was judged as low risk of selection of bias (87.5%), with two studies being judged as unclear, due to unclarity on the type (i.e., content) of training sessions practiced per week. For most of the studies (*n* = 14) (87.5%) displayed unclear risk of bias to “selective outcome reporting” domain, because the studies did not clearly describe the exact number of players considered for the respective statistical analyses. No studies were judged with high risk of bias for each domain.

### Training load quantification methods

Regarding ETL during training sessions (Table 2), eight studies have adopted speed-based intensity zones using arbitrary/fixed thresholds (between 12.2 and 18 km⋅h^–1^) (Mara et al., 2015a,b; Clemente et al., 2019; Costa et al., 2019a; Douchet et al., 2021; Doyle et al., 2021; Romero-Moraleda et al., 2021; Xiao et al., 2021), while one study considered individual fitness level (Scott and Lovell, 2018). Two studies reported that players covered greater total distance and high-speed distance (>12.2 km⋅h^–1^) during the pre-season compared to early competitive season, and then decreased late in the season (Mara et al., 2015b; Clemente et al., 2019). On the other hand, three studies reported that total distance and high-speed distance (>12.6 km⋅h^–1^ and >maximal aerobic speed [MAS]) were stable in training sessions during international tournaments, independently of the data reported for official matches (Scott and Lovell, 2018; Costa et al., 2019a; Doyle et al., 2021).

**TABLE 2:** Training load data during training sessions in highly trained and elite adult women soccer players.

Reference	Training load variables	Values	Description
Clemente et al., 2019	TD (m) HSD > 14 km⋅h^–1^ (m) Sprint distance > 20 km⋅h^–1^ (m)	3000 to 5000 200 to 400 1000 to 1500	Mean of weekly load (lowest to highest load values) during 5 weeks of the pre-season.
Costa et al., 2018a	HR_*ex*_ (%HR_*peak*_) TRIMP (AU) s-RPE (AU)	70 ± 3 to 75 ± 4 72 ± 18 to 138 ± 29 193 ± 60 to 442 ± 159	Mean ± standard deviations (lowest to highest load values) during 3 weeks of the competitive season.
Costa et al., 2018b	HR_*ex*_ (bpm) TRIMP (AU) s-RPE (AU)	138 ± 13 to 149 ± 16.8 77 ± 36 to 110 ± 31 281 ± 117 to 369 ± 111	Mean ± standard deviations (lowest to highest load values) during 1 week of the competitive season.
Costa et al., 2019a	TD (m) HSD > 12.6 km⋅h^–1^ (m) s-RPE (AU)	2201 to 4284 130 to 756 131 to 360	Median (lowest to highest load values) during 9 days of international tournament.
Costa et al., 2019b	HR_*ex*_ (%HR_*peak*_) TRIMP (AU) s-RPE (AU)	74 ± 2 192 ± 21 326 ± 33	Mean ± standard deviations values during 6 weeks of the competitive season.
Costa et al., 2019c	HR_*ex*_ (bpm) TRIMP (AU) %HR_*peak*_ Time > 90 of %HR_*peak*_ (min) s-RPE (AU) RPE (AU)	139 ± 12 211 ± 81 73 ± 6 8 ± 7 338 ± 107 3 ± 2	Mean ± standard deviations values during 6 weeks of the competitive season.
Costa et al., 2021a	s-RPE (AU)	604 ± 70	Mean ± standard deviations value during 4 weeks of pre-season.
Costa et al., 2021b	HR_*ex*_ (bpm) TRIMP (AU) %HR_*peak*_ s-RPE (AU)	143 to 147 187 to 189 75 to 77 377 to 411	Mean values during 14 days of the competitive season.
Douchet et al., 2021	TD (m) HSD > 18 km⋅h^–1^ (m) Number of sprints > 19.4 km⋅h^–1^(counts) Accelerations > 2 m⋅s^–2^ (counts) Decelerations > −2 m⋅s^–2^ (counts) HR_*ex*_ (%) s-RPE (AU) RPE (AU)	3870 ± 870; 5090 ± 620 124 ± 61; 155 ± 92 7 ± 3; 7 ± 3 28 ± 12; 56 ± 10 31 ± 12; 61 ± 14 63 ± 6; 67 ± 7 201 ± 47; 357 ± 50 3 ± 1; 5 ± 1	Mean ± standard deviations values during 2 weeks: Low week load (1 week); heavy week load (1 week).
Doyle et al., 2021	TD (m) HSD > 12.6 km⋅h^–1^ (m) Number of sprints > 19.4 km⋅h^–1^(counts) Accelerations > 3 m⋅s^–2^ (counts) Decelerations > −3 m⋅s^–2^ (counts) s-RPE (AU) RPE (AU)	3339 to 59335 58 to 389 2 to 14 28 to 56 21 to 46 203 to 721 3 to 7	Median (lowest to highest load values) during 1 week of the competitive season.
Fernandes et al., 2021	RPE (AU)	3 to 6	Mean of weekly load (lowest to highest load values) during 10 weeks of the competitive season.
Mara et al., 2015a	TD (m) HSD > 12.2 km⋅h^–1^ (m) Sprint distance 19.4 km⋅h^–1^ (m) Accelerations > 2 m⋅s^–2^ (counts) Decelerations > −2 m⋅s^–2^ (counts)	6581 ± 847 880 ± 244 333 ± 107 49 ± 13 18 ± 9	Mean ± standard deviations values during 1 week of the pre-season.
Mara et al., 2015b	TD (m) HSD > 12.6 km⋅h^–1^ (m) Number of sprints > 19.4 km⋅h^–1^(counts) Accelerations > 2 m⋅s^–2^ (counts) Decelerations > −2 m⋅s^–2^ (counts)	6646 ± 111; 5437 ± 106 1415 ± 42; 1027 ± 40 27 ± 15; 24 ± 9 56 ± 19; 49 ± 14 22 ± 10; 20 ± 10	Mean ± standard deviations values during 18 weeks: pre-season (6 weeks); competitive season (12 weeks).
Romero-Moraleda et al., 2021	TD (m) HSD > 15 km⋅h^–1^ (m) Accelerations > 1 m⋅s^–2^ (counts) Decelerations > −1 m⋅s^–2^ (counts) s-RPE (AU) RPE (AU)	2496 ± 1639 to 4975 ± 1319 170 ± 214 to 494 ± 248 70 ± 56 to 144 ± 39 17 ± 17 to 38 ± 16 167 ± 134 to 579 ± 139	Mean ± standard deviations (lowest to highest load values) during 5 months of the competitive season.
Scott and Lovell, 2018	HSD > 12.6 km⋅h^–1^ (m) Minutes spent > 80 %HR_*m*á*x*_ (min) TRIMP (AU) RPE (AU)	250 to 2500 5 to 65 150 to 400 3 to 8	Individual means (lowest to highest load values) during 21 days of the competitive season.
Xiao et al., 2021	TD (m) HSD > 12.9 km⋅h^–1^ (m)	3662 to 18461 1173 to 4994	Mean accumulated workloads over 4 weeks of the competitive season.

HR, heart rate; HSD, high-speed distance; s-RPE, session-rating of perceived exertion; TD, total distance; TRIMP, training impulse.

Internal training load was quantified using HR- and RPE-based methods (Table 2). Seven studies quantified ITL using HR (Costa et al., 2018a,b, 2019b,2019c,2021b; Scott and Lovell, 2018; Douchet et al., 2021). Seven studies individualized physiological responses to exercise relative to HR_*max*_ obtained by an incremental protocol until exhaustion Costa et al., 2018a,b, 2019b,2019c, 2021a,b; Scott and Lovell, 2018). Six studies quantified ITL using RPE (Scott and Lovell, 2018; Costa et al., 2019c; Douchet et al., 2021; Doyle et al., 2021; Fernandes et al., 2021; Romero-Moraleda et al., 2021), while ten studies reported s-RPE (Costa et al., 2018a,b, 2019a,2019b,2019c, 2021a,b; Douchet et al., 2021; Doyle et al., 2021; Romero-Moraleda et al., 2021).

### Training load and fatigue

The relationship between TL and fatigue has been examined in eight studies (Mara et al., 2015b; Costa et al., 2018a,2019a,2019b,2021b; Scott and Lovell, 2018; Douchet et al., 2021; Fernandes et al., 2021). During a 9-day international tournament, no significant within-subject correlations were observed between post-training night sleep parameters (e.g., total sleep time and sleep efficiency) and ETL metrics (e.g., distance and high-speed distance) (Costa et al., 2019a). On the other hand, small to moderate (*r* = −0.43 to −0.17) within-subject correlations were observed between ITL (s-RPE and training impulse [TRIMP]) and sleep parameters (sleep duration and efficiency) during a 14-day competitive period (Costa et al., 2021b). Moreover, significant differences in sleep patterns and autonomic nervous activity responses when night training sessions were compared to competitive day matches and rest days, suggesting that the time of day for soccer practice may disrupt sleep patterns and nocturnal autonomic activity (Costa et al., 2018a,2019b). In addition, Douchet et al. (2021) showed that a week with more accelerations and decelerations were significantly associated (*r* = 0.94) with increased fatigue as witnessed by the greater RPE and perceived well-being (i.e., Hooper index). Associations were also found between perceived well-being (i.e., stress and fatigue) and s-RPE (*r* = 0.69) during a 10-week competitive period (Fernandes et al., 2021).

Self-reported measures of fatigue have shown significant associations with ELT (e.g., high-speed distance) on the previous day during a tournament (Scott and Lovell, 2018). Scott and Lovell (2018) described that self-reported fatigue and muscle soreness were negatively associated (small magnitude) with high-speed distance covered (*r* = −0.20) using either fixed (>12.6 km/h^–1^) or individual thresholds during a 21-day training camp. Finally, Mara et al. (2015b) showed that self-reported fatigue and sleep times were not correlated with the total distance covered >12.6 km⋅h^–1^, whereas muscle soreness was negatively correlated (moderate magnitude) with ETL parameters during the pre-season.

### Training load and training adaptations

Information of the dose–response relationship between TL and training adaptations in highly trained and elite adult women soccer players is limited to one study (Mara et al., 2015b). Positive correlations were reported between changes in intermittent endurance capacity assessed through performance in the Yo-Yo Intermittent Recovery Test – level 2 after the pre-season (*r* = 0.70), and accumulated ETL (*r* = 0.71; *r* = 0.56, respectively), high-speed distance (>12.6 km⋅h^–1^) and accelerations (>2 m⋅s^–2^) during the pre-season (Mara et al., 2015b).

### Training load and injury

Only one study reported the relationship between TL and injuries (defined as an event that caused the player to miss at least 1 subsequent practice or match and lower extremity injuries) in women soccer players (Xiao et al., 2021), revealing that players that sustained an injury had significantly higher 2-, 3-, and 4-week accumulated TL and total distance covered as compared with injury-free players during the same time frame.

## Discussion

In the current systematic review, we confirmed the limited information available about training outcomes in highly trained and elite adult women soccer players, especially in the relationship between TL, training adaptation and injuries (Kirkendall and Krustrup, 2021). Additionally, current monitoring practices in highly trained and elite adult women soccer players are sparse, which underline the need for conducting studies or surveys based on that implemented in men’s soccer (Akenhead and Nassis, 2016).

### External training load monitoring

Wearable microtechnology incorporating GPS, local positioning systems or triaxial accelerometers have shown good ability to measure ETL based on distance, speed, and accelerations in team sports (Scott et al., 2016; Torres-Ronda et al., 2022). In this context, one key aspect of training prescription is to understand how the individual athlete is coping with the imposed training demands. While the use of individualized intensity zones to quantify ITL (e.g., based on HR_*max*_ or HR_*reserve*_) is widely adopted among sports practitioners, especially in men’s soccer (Dellal et al., 2012; Akenhead and Nassis, 2016), the use of individualized intensity zones for ETL quantification (based on speed and acceleration) is not fully established, especially in women’s soccer. Actually, the individualization of speed-based EL based on testing metrics (e.g., MAS; and maximal sprinting speed, MSS) has received increased attention in adult men (Hunter et al., 2015; Rago et al., 2019c,^2020^) and youth soccer (Mendez-Villanueva et al., 2013; Abbott et al., 2018) players, but not in women players. Generally, match-analysis reports in women’s soccer described physical match data using two different sprint thresholds, based on either fixed (20 km⋅h^–1^) and individualized (90% mean speed obtained from a 20-m sprint test) speed zones (Nakamura et al., 2017). Similar patterns were observed between halves, and playing positions, but fixed speed zones may have likely underestimated the mean duration, distance, and the number of sprint sequences (Nakamura et al., 2017). Additionally, only one study employed individual speed zones based on MAS and MSS, showing that individualizing ETL metrics did not improve the relationship between training load and self-reported fatigue (Scott and Lovell, 2018). In this study (Scott and Lovell, 2018), HSD > 12.6 km⋅h^–1^ ranged between 250 to 2500 m during 21 days of the competitive season. However, the latter study employed MAS and MSS separately, adopting the following criteria: distance covered >80% MAS, MAS, >50% MSS and 65% MSS (Scott and Lovell, 2018). Moreover, it is important to note the use of 50–65% MSS that could be close to the potential MAS (15–18 km⋅h^–1^) in elite athletes (Rago et al., 2020), considering that women soccer players peak approximately at 30–32 km⋅h^–1^ during a match (Datson et al., 2014, 2017). This assumes a linear relationship between aerobic (MAS) and anaerobic (Li et al., 2019) power that may consequently result in an erroneous interpretation of ETL. In this context, once MAS and MSS have been obtained from incremental and 40-m sprint tests, respectively, suggested by the assessment of anaerobic speed reserve (ASR) (Bundle et al., 2003). The use of ASR rely on the fact that different players with the same MAS, but different sprinting capacity, require different training prescription when exercising at intensities above MAS (Buchheit and Laursen, 2013). However, no information is available regarding ASR-based training load in women’s soccer. Similarly, no data are available on the individual training prescriptions based on maximal acceleration capacity in women soccer players. This might be relevant due to the frequent acceleration demands required during soccer training and match play, as well as the variations in acceleration capacity observed throughout different periods of the season (Mara et al., 2015b).

### Internal training load monitoring

Internal training load is usually quantified using HR monitors, which generically provide information about the aerobic contribution during exercise (Achten and Jeukendrup, 2003). A potential strength of HR-based methods is the information about aerobic contribution based on the strong relationship with oxygen consumption during exercise, when data are expressed as percentage of HR_*max*_ or HR_*reserve*_ (Achten and Jeukendrup, 2003). On the other hand, a potential limitation of HR-based methods is the failure to detect anaerobic-oriented efforts such as sudden sprints or explosive bursts commonly observed during soccer training and match-play (Achten and Jeukendrup, 2003; Dellal et al., 2012). Therefore, an integrated approach encompassing both ETL and ITL is imperative to provide a full picture the exercise demands placed on the athletes. Nonetheless, HR-based variables are sensitive in detecting day-to-day variations in TL in highly trained and elite adult women soccer players under different competitive conditions, such as a domestic league competitive period (Costa et al., 2018a) or an international tournament (Scott and Lovell, 2018).

Beyond the usefulness of HR-based methods, and despite the development of women’s soccer, most women’s teams worldwide might still have weak budgets compared to that of men’s teams to acquire sophisticated equipment, which frequently results in adopting cost-free methods based on the post-training subjective RPE (Costa et al., 2019c). The use of RPE-based methods such as the s-RPE is deemed to be valid in women soccer players based on its large relationship with HR-based methods (e.g., training impulse, Edwards’ TL; Costa et al., 2019c). The latter study (Costa et al., 2019c), found an large correlation between s-RPE with TRIMP (*r* = 0.74–0.82), with TRIMP values ranging from 211 ± 81 AU and s-RPE values from 388 ± 107 AU. However, the connection between RPE-based and ETL parameters is unknown in women soccer players. This would be useful to discriminate between TL parameters, providing practitioners with evidence-based TL metrics to be adopted in their monitoring systems.

### Fatigue

Fatigue has been defined as the inability to complete a task that was once achievable within a recent time frame (Pyne and Martin, 2011). Acute (immediately after) and residual (up to 72 h) fatigue may temporarily impair players’ readiness to train and compete (Silva et al., 2018). In this context, monitoring TL may be useful to infer about acute and residual fatigue, allowing individual adjustments to training programs, improve well-being, restore physical capacity, and inform about the recovery process (Hader et al., 2019). Generally, women soccer players may need up to 72 h to achieve full neuromuscular recovery after a competitive match (Andersson et al., 2008; Krustrup et al., 2010; Sjokvist et al., 2011). Specifically, sprint performance, countermovement jump (CMJ), and peak torque in knee extension and flexion are reduced after a match (Andersson et al., 2008; Krustrup et al., 2010; Sjokvist et al., 2011). However, changes in neuromuscular function following a match and throughout the recovery period need further elucidation in women’s soccer.

Notably, self-reported measures of fatigue are widely accepted among practitioners due to their ease to use and low-cost (Hooper et al., 1995). Indeed, subjective measures have shown acceptable sensitivity and consistency in athletes (Saw et al., 2016). For instance, self-reported measures of fatigue have shown significant associations with GPS-based TL on the previous day during a tournament (Scott and Lovell, 2018). In this study (Scott and Lovell, 2018), irrespective of the quantification method adopted for HSD > 12.6 km⋅h^1^ and very HSD (fixed or individualized speed zones), negative small correlations were observed with fatigue and soreness (*r* = −0.25 to −0.14). Actually, it seems that self-reported outcomes might be dependent on the training and competitive context, underlining the need to consider studies with more extensive periods (e.g., full or multiple seasons) and over a wide range of fatigue and recovery indicators; specially because there is very limited information about acute and residual fatigue in relation to TL in highly trained and elite adult women soccer players. Findings in men’s soccer have shown significant correlations between various indicators of acute and residual fatigue and TL (Thorpe et al., 2017a; Hader et al., 2019). Specifically, non-invasive measures of fatigue such as sitting HR, submaximal HR, CMJ and self-reported questionnaires have shown responsiveness to daily and acute changes in TL over time (Thorpe et al., 2015, 2017b). These measures can be routinely applied to a number of athletes to monitor changes in training status (Buchheit, 2014). Moreover, subjective measures of fatigue can be easily incorporated into the monitoring systems, with the advantage of being cost-free and showing responsiveness to TL (Saw et al., 2016).

Information regarding HR measures during recovery after training sessions or matches in highly trained and elite adult women’s soccer has been predominantly conducted during sleep time (Costa et al., 2018a,b, 2019a,2019b,2021b). Most players from the same team presented fluctuations in nocturnal cardiac autonomic activity (i.e., coefficient of variation ranging from 2.8 to 9.0%) (Costa et al., 2019a). However, no within-subject associations over time were observed between TL (e.g., s-RPE, TRIMP and distance > 12 km⋅h^–1^) and HR parameters during sleep (Costa et al., 2019a,2021b). The authors (Costa et al., 2019a,2021b) suggested that the amount of training and match demands (s-RPE ranging between 348 to 690 AU and TRIMP between 191 to 247 AU) prescribed to the players was not high enough to cause meaningful changes in cardiac sympathetic and parasympathetic activities during sleep. Additionally, no evidence is available about resting and submaximal HR in women soccer players, as previously described in men’s soccer (Naranjo et al., 2015). However, women soccer players showed significant differences in sleep patterns and autonomic nervous activity responses when night training sessions were compared to competitive day matches and rest days, suggesting that the time of day for soccer practice may disrupt sleep patterns and nocturnal autonomic activity (Costa et al., 2018a,2019b). For example, during a 9-day international tournament, no significant within-subject correlations were observed between post-training night sleep parameters (sleep time, sleep efficiency and heart rate variability during sleep) and both ETL (i.e., HSD > 12.6 km⋅h^1^ ranging between 130 to 756 m) and s-RPE (i.e., ranging between 131 to 360 AU) (Costa et al., 2019a). On the other hand, small within-subject correlations were observed between ITL (s-RPE [ranging between 377 to 411 AU] and TRIMP [ranging between 187 to 189 AU]) and sleep parameters (sleep duration and efficiency) during a 14-day competitive period (Costa et al., 2021b). Thus, even under stress imposed by tournament scheduling and training and match loads, the players maintained relatively good consistency in sleep habits to recover from the training sessions and matches.

### Training adaptations

Following an acute fatigue phase, it is expected that chronic exposure to TL contributes to benefits in players’ fitness levels, resulting in positive health or performance adaptations (Mara et al., 2015b). Thus, understanding changes in physiological and functional capacities of women soccer players is of upmost importance given its meaningful connection with physical performance during a match (Krustrup et al., 2005). Currently, the effectiveness of various training interventions (e.g., interval, resisted sprint, and plyometric training) in women’s soccer is well-documented (Datson et al., 2014). However, it is important to note that some coaches could prescribe training programs based on their professional and educational background, with less attention to published training interventions (Rago, 2020). This could be due to the fact that evidence-based analytic drills do not always fit within the technical staff philosophy. In this sense, observational studies considering the exercise prescribed by coaches and the associated training outcomes could aid in understanding the effectiveness of training programs without the need to design intervention studies. For example, employing field performance tests at different seasonal points while simultaneously quantifying TL allows the computation of the relationship between TL and changes in performance (Jaspers et al., 2017). To date, only one study has adopted an observational design based on fitness testing at different seasonal points (i.e., 6 weeks of pre-season and 12 weeks of the competitive season) concerning TL; positive correlations were reported between changes in intermittent endurance capacity and ELT (Mara et al., 2015b). However, the latter study did not consider the individual capacity to adjust TL and only included maximal tests (e.g., jump, sprint, and time-to exhaustion). In this context, non-invasive measures of resting or submaximal HR may aid in detecting training adaptations without having the athletes to perform until exhaustion (Buchheit, 2014; Naranjo et al., 2015; Rago et al., 2019d). Also, different force-time and force-velocity components during the different phases of a jump might be sensitive in detecting training-induced changes (Gathercole et al., 2015). In summary, information regarding the dose–response relationship between TL and training adaptations in highly trained and elite adult women soccer players is limited to one study (Mara et al., 2015b), which did not consider the individual capacity to quantify TL. Thus, further studies are warranted to explore the dose–response relationship that may elicit the desired long-term performance outcomes.

### Injuries

It has been reported that players might sustain illnesses or time-loss injuries during the season (Fuller et al., 2006). Injury incidence in women’s soccer ranged between 1.2–7.0 injuries per 1,000 training hours, and 12.6–24.0 per 1,000 match hours (Giza et al., 2005; Jacobson and Tegner, 2007; Tegnander et al., 2008; Alahmad et al., 2020). The latter studies were based on descriptive epidemiological information, without inferences computed on injury incidence or risk in relation to training outcomes. Only one study reported information about the relationship between TL and injuries. Xiao et al. (2021) found that higher accumulated player load and total distance covered (values ranging between 3662 to 18461 m) were associated with injury in women soccer players during the same time frame. Thus, it is currently not possible to provide an explanation about training-related factors associated to injury or medical assistance. Alternatively, registering the occurrence of medical assistance (instead of time-loss injuries), as previously described in other team sports, could be of interest (Martinez-Riaza et al., 2017). Indeed, erroneous training progressions could result in delayed muscle soreness (Thorpe et al., 2017a) with an associated search for medical assistance. In general, the connection between training outcomes and injury risk has yet to be explored in women soccer players.

## Conclusion

Information about TL in women soccer players is very sparse. Thus, it is very difficult for practitioners to consider evidence-based practices for training sessions beyond solid information available from match-analysis studies. For example, further studies on training contents, loads and adaptations are warranted to design match-like practice sessions and drills for women’s soccer. Moreover, the dose–response relationships between TL, fatigue, training adaptions and injuries need to be clarified to understand the optimal training stimulus to enhance performance while preserving players’ health. Also, future studies should encompass extensive periods with different seasonal phases (e.g., off-season, pre-season and in-season) and fixtures (e.g., ordinary microcycles, congested periods, national team breaks) with special emphasis on how TL affects training outcomes (e.g., acute fatigue, training adaptations, and injury risk) as previously examined in men’s soccer (Jaspers et al., 2017). In addition, as future research, would be imperative to understand the importance of training load prescription and adaptation within youth women’s soccer players from different competitive levels, which may help to preventing decrements in performance, or enhancing recovery in women’s soccer population.

We have attempted to summarize current TL monitoring during training sessions in women’s soccer, which may help to inform the practitioners working with highly trained and elite players, but also identify knowledge gaps and make suggestions for future research. More specifically, from a physical and physiological perspective, future research should use monitoring technology to determine more accurately the physical and physiological demands of training in women soccer players.

## Data availability statement

The original contributions presented in this study are included in the article/Supplementary material, further inquiries can be directed to the corresponding author.

## Author contributions

JC, VR, and JB contributed to the conceptualization and methodology. JC, VR, PB, EA, and JB contributed to the formal analysis and investigation. JC, VR, PB, and EA contributed to the data curation. VR, PF, AS, EA, and JB contributed to the writing—review and editing. JC and JB contributed to the writing—original draft preparation and visualization. JC contributed to the software. JB contributed to the validation, resources, supervision, and project administration. All authors read and agreed to the published version of the manuscript.
