# Supplementary material for: Training in women soccer players: A systematic review on training load monitoring
Source: Front Psychol. 2022 Jul 29;13:943857. doi: 10.3389/fpsyg.2022.943857 (PMC9372388; doi:10.3389/fpsyg.2022.943857)
Supplement: Supplementary file 1 [file Table_1.docx]

| **Supplementary Table S1.** Qualitative assessment domains criteria used for training load monitoring in highly trained and elite adult women’s soccer studies judgment. | | | | | | | | | | | | | | | | | |
| --- | --- | --- | --- | --- | --- | --- | --- | --- | --- | --- | --- | --- | --- | --- | --- | --- | --- |
| **Study** | **1** | **2** | **3** | **4** | **5** | **6** | **7** | **8** | **9** | **10** | **11** | **12** | **13** | **14** | **15** | **16** | **total %** |
| (Clemente et al., 2019) | 1 | 1 | 1 | 1 | 0 | 1 | 1 | 1 | 1 | 1 | 1 | 1 | n/a | 1 | 1 | 1 | 93.3 |
| (Costa et al., 2018a) | 1 | 1 | 1 | 1 | 0 | 1 | 1 | 1 | 1 | 1 | 1 | 1 | n/a | 1 | 1 | 1 | 93.3 |
| (Costa et al., 2018b) | 1 | 1 | 1 | 1 | 0 | 1 | 1 | 1 | 1 | 1 | 1 | 1 | n/a | 1 | 1 | 1 | 93.3 |
| (Costa et al., 2019a) | 1 | 1 | 1 | 1 | 0 | 1 | 1 | 1 | 1 | 1 | 1 | 1 | n/a | 1 | 1 | 1 | 93.3 |
| (Costa et al., 2019b) | 1 | 1 | 1 | 1 | 0 | 1 | 1 | 1 | 1 | 1 | 1 | 1 | n/a | 1 | 1 | 0 | 86.7 |
| (Costa et al., 2019c) | 1 | 1 | 1 | 1 | 0 | 1 | 1 | 1 | 1 | 1 | 1 | 1 | n/a | 1 | 1 | 1 | 93.3 |
| (Costa et al., 2021a) | 1 | 1 | 1 | 1 | 1 | 1 | 1 | 1 | 1 | 1 | 1 | 1 | n/a | 1 | 1 | 1 | 100.0 |
| (Costa et al., 2021b) | 1 | 1 | 1 | 1 | 0 | 1 | 1 | 1 | 1 | 1 | 1 | 1 | n/a | 1 | 1 | 1 | 93.3 |
| (Douchet et al., 2021) | 1 | 1 | 1 | 1 | 0 | 1 | 1 | 1 | 1 | 1 | 1 | 1 | n/a | 1 | 1 | 1 | 93.3 |
| (Doyle et al., 2021) | 1 | 1 | 1 | 1 | 0 | 1 | 1 | 1 | 1 | 1 | 1 | 1 | n/a | 1 | 1 | 1 | 93.3 |
| (Fernandes et al., 2021) | 1 | 1 | 1 | 1 | 0 | 1 | 1 | 1 | 1 | 1 | 1 | 1 | n/a | 1 | 1 | 1 | 93.3 |
| (Mara et al., 2015a) | 1 | 1 | 1 | 1 | 0 | 1 | 1 | 1 | 1 | 1 | 1 | 1 | n/a | 1 | 1 | 1 | 93.3 |
| (Mara et al., 2015b) | 1 | 1 | 1 | 1 | 0 | 1 | 1 | 1 | 1 | 1 | 1 | 1 | n/a | 1 | 1 | 0 | 86.7 |
| (Romero-Moraleda et al., 2021) | 1 | 1 | 1 | 1 | 0 | n/a | 1 | 1 | 1 | 1 | 1 | 1 | n/a | 1 | 1 | 1 | 92.9 |
| (Scott and Lovell, 2018) | 1 | 1 | 1 | 1 | 0 | n/a | 1 | 1 | 1 | 1 | 1 | 1 | n/a | 1 | 1 | 1 | 92.9 |
| (Xiao et al., 2021) | 1 | 1 | 1 | 1 | 1 | n/a | 1 | 1 | 1 | 1 | 1 | 1 | n/a | 1 | 1 | 1 | 100.0 |

Note: 1) clarity of purpose; 2) relevance of background literature; 3) appropriateness of the study design; 4) study sample; 5) sample size justification; 6) informed consent (if any); 7) outcome measures – reliability; 8) outcome measures – validity; 9) detailed methods description; 10) significance of results reporting; 11) analysis methods; 12) practical importance; 13) description of drop-outs (if any); 14) appropriately conclusions; 15) practical implications; 16) study limitations. A binary scale was used to score these items (1=yes; 0=no), except for items 6) and 13), which could also be classified as not applicable (n/a).
