# Supplementary material for: Training in women soccer players: A systematic review on training load monitoring
Source: Front Psychol. 2022 Jul 29;13:943857. doi: 10.3389/fpsyg.2022.943857 (PMC9372388; doi:10.3389/fpsyg.2022.943857)
Supplement: Supplementary file 2 [file Table_2.docx]

**Supplementary Table S2** – RoBANS domains criteria used for training load monitoring in elite women’s soccer studies judgment.

| **Domain** | **Description** |
| --- | --- |
| **D1: Selection of participants** | **Selection bias** caused by inadequate selection of participants.  Elite women’s soccer (i.e., player's competing at the international leagues/tournaments; player's competing in national and/or state leagues/tournaments; individuals on a national team). |
| **D2: Confounding variables** | **Selection bias** caused by inadequate confirmation and consideration of confounding variable.  As confounding variables (if not controlled for), we consider the lack of information regarding number, volume and type (i.e., content) of training sessions per week. |
| **D3: Exposure measurement** | **Training load bias** caused by inadequate measurement of exposure.  Outcome data measurement (i.e., the method of training load measurement) should be described in sufficient detail to allow for replication and the main outcome measures should be collected with a "standardised" approach. Objective data recorded with an equipment should provide the error reliability data. |
| **D4: Blinding outcome assessment** | **Detection bias** caused by inadequate blinding of outcome assessment.  Task assessor and/or data analysist should be blinded to group if objective data (collected by person). If it is objective machine-based collection (e.g., heart rate monitoring), no blinding is needed. If the subjects are blinded for data results of the study. |
| **D5: Incomplete outcome data** | **Attrition bias** caused by inadequate handing of incomplete data outcome.  When missing data is > 5% for training load data, or there is a loss of follow-up > 5%. |
| **D6: Selective outcome reporting** | **Reporting bias** caused by selective outcome reporting.  Based on reporting of the collected training load measures.  Outcomes not described the exactly number of player's (n) that were considered for the respective statistical analyses. |
